# Indigenous Motherwork in Crisis: Caregiving, Resistance, and Community Survival During the COVID-19 Pandemic

**DOI:** 10.1007/s11199-025-01584-4

**Published:** 2025-04-21

**Authors:** Rory Glover, Margaret Mary Downey, Catherine E. O’Connor, Michelle Johnson-Jennings, Kristi Ka’apu

**Affiliations:** 1 Tulane University, New Orleans, LA USA; 2 Tulane University School of Social Work, 127 Elk Place, #8906, New Orleans, LA 70112-2699 USA; 3 University of Washington, Indigenous Wellness Research Institute, Seattle, WA USA

**Keywords:** Indigenous Motherwork, Indigenous, Motherwork, Maternal Labor, COVID-19, Caregiving

## Abstract

This study describes the lived experiences of Indigenous mothers during the COVID-19 pandemic, focusing on caregiving strategies, resilience, and leadership within their families and communities while navigating systemic gendered and racial inequalities. The COVID-19 pandemic amplified long-standing structural inequities, disproportionately impacting Indigenous communities. As primary caregivers and cultural stewards, Indigenous mothers faced intensified burdens, including economic precarity, expanded domestic labor, and systemic exclusions. Their caregiving, however, functioned as a site of resistance, reinforcing intergenerational survivance and asserting Indigenous sovereignty through relational care. A total of 31 critical ethnographic interviews were conducted with Indigenous mothers following an Indigenous research framework. Interviews explored survival strategies, power dynamics, and responsibilities amid the COVID-19 pandemic. Emergent themes included prioritizing survival needs, navigating institutional barriers, and exercising cultural resilience in caregiving. Despite increased family community and sociopolitical stressors, Indigenous mothers demonstrated adaptability, leadership, and collective strength. Indigenous mothers demonstrated resilience and resourcefulness in sustaining their families and communities amid the COVID-19 crisis. Their experiences underscore the need for culturally responsive interventions and policies that recognize and support Indigenous mothering as a foundation for community well-being. Findings highlight the urgency of policy reforms, targeted resource allocation, and culturally affirming support systems that empower Indigenous mothers as leaders and caregivers. Investing in their well-being strengthens entire communities.

This study explored nuanced experiences of Indigenous mothers (i.e., American Indians and Alaska Natives) during the COVID-19 global pandemic. Enduring colonialism amplified Indigenous vulnerability to COVID-19 and disproportionately impacted Indigenous populations, particularly in terms of infection rates, hospitalizations, and fatalities (Connolly et al., 2021; Fortuna et al., 2020; Hornback & Ramos, 2021; Leggat-Barr et al., 2021). Indigenous mothers hold a pivotal role within these communities, traditionally revered as the cornerstone of families and communities (Baskin, 2020; Lajimodiere, 2011; McKinley, 2023a, b; Udel, 2001). Amid the pandemic, we found Indigenous mothers faced added strains, bearing the burden of restricted childcare and economic resources while shouldering significant emotional and mental labor as frontline caregivers within the family and the broader communities. Despite the recognition of Indigenous mothers as central figures in their families and communities, their experiences and unique challenges during the pandemic remain underexplored.

Drawing upon existing literature, we aimed to illuminate the intersection of motherwork with traditional Indigenous concepts of mothering, providing insights into how Indigenous mothers have navigated the unprecedented challenges brought forth by the pandemic. Dean et al. (2022) identified the mental load (or overload) of mothers’ caregiving roles during the pandemic, highlighting the taxing interplay of cognitive and emotional labor characterized as invisible, boundaryless, and enduring—often with detrimental implications for health and well-being. Although research on the gendered dynamics of childcare and employment during COVID-19 has garnered attention (Carlson et al., 2022; Collins et al., 2021; Craig & Churchill, 2021), a noticeable gap in understanding the specific impacts on Indigenous mothers exists in broader discourse.

We sought to address this gap by examining how Indigenous mothers engage in motherwork as a means of survival, empowerment, and identity amid the COVID-19 pandemic. We aimed to contribute to the growing body of literature on Indigenous motherhood, offering insights that can inform interventions and policies to support Indigenous communities during and beyond the COVID-19 crisis. We explored the lived experiences of Indigenous mothers from a Southeastern tribe during the COVID-19 pandemic. First, we explored the intersection of motherwork with traditional Indigenous concepts of mothering. Next, we analyzed the pandemic’s specific impact on Indigenous communities motherwork’s role in buffering these impacts. Finally, we concluded that Indigenous motherwork is essential care work that sustains Indigenous communities. We considered the immediate needs of Indigenous mothers and hope to contribute to a broader understanding of the resilience and strength of Indigenous communities. By centering Indigenous perspectives and experiences, our goal was to enhance understanding of the COVID-19 pandemic’s impacts on diverse families and communities. Ultimately, we advocated for culturally responsive interventions and policies specifically aimed at supporting and reinforcing the strength of Indigenous mothers, who bear the weight of upholding their communities despite being disproportionately by social, financial, and familial responsibility.

## Conceptual and Theoretical Frameworks on Motherwork and Resilience

Three key conceptual frames guided our analysis: motherwork; the framework of historical oppression, resilience, and transcendence (FHORT); and reproductive justice. Here, we outline each conceptual frame’s influence on our study. First, our analysis was underpinned by Collins’s (1987, 1997) seminal work on motherwork as it centers social context, emphasizing systemic racial oppression and economic exploitation. For Collins, mothers of color navigate entwined struggles for control over their bodies, their children’s upbringing, and their negotiation of self-definitions as mothers. This framework was salient for our analysis of Indigenous mothering during the COVID-19 global pandemic, as it focuses on survival, power, and identity and emphasizes the essential work mothers perform for their children’s survival in oppressive systems (Collins, 1987, 1997).

Motherwork also includes caring for other children in the community and future generations in extended family and kinship networks (Liddell et al., 2021). Like other Black feminists, Collins’s concept of motherwork extends gender theorization beyond the traditional boundaries of the white, capitalist, nuclear family to encompass the multifaceted labor of nurturing, resistance, and community building carried out by Black women (Collins, 1987). She describes motherwork as part of all marginalized women’s experiences of mothering, including Indigenous mothers (Liddell et al., 2021; McKinley, 2023a, b; Udel, 2001).

Indigenous motherwork differs from patriarchal Western ideals (e.g., independence, privacy, domination of children) by blurring boundaries between public and private spheres and individual and collective identities, fostering interdependence and mutual family support. In Indigenous mothering ideology, children are seen as independent spirits with valuable lessons to impart to their caregivers (Simpson, 2006), an egalitarian family structure emphasizing reciprocal relationships. These practices extend beyond the biological family to encompass the broader community and the spiritual dimensions of Indigenous collective life (Anderson, 2011, 2016; Takševa, 2017). Udel (2001) emphasized culturally distinct characteristics of Indigenous motherwork, stating, “Native women’s motherwork, in its range and variety, is one form of this activism, an approach that emphasizes Native traditions of ‘responsibilities’ as distinguished from Western feminism’s notions of ‘rights’” (p. 43).

Crucially, motherwork identifies labor outside the home or domestic sphere as a necessary and generative part of mothering. Motherwork is thus uniquely relevant to Black and Indigenous mothers who have often labored outside the home, often in extreme conditions (e.g., exploited, enslaved, precarious), to provide for their families. Both Black and Indigenous women have long been excluded from public and private resources that support white, middle-class mothers, such as “mothers pensions” (i.e., the predecessor to welfare) or charitable donations from white churches or institutions. Additionally, motherwork emphasizes how mothers of color often engaged in paid caretaking of white children while continuing the unpaid nurturing and emotional labor to support their own families, even extending beyond biological motherhood to encompass communal mothering (Bell-Scott et al., 1991; Collins, 1997; James, 1993). Communal mothering has emerged as a powerful form of political resistance within the lives of Black and Indigenous women, with their caregiving roles as a form of social and political labor that contributes to community resilience and empowerment in the face of crises.

This multifaceted concept encompasses daily caregiving tasks, emotional and mental labor, household management, education, advocacy, nurturing, cultural stewardship, and the physical and emotional care of children and the community. Collins (1997) articulated how colonization, racism, and sexism have given rise to a distinct motherhood ideology for women of color due to the lack of privileges assumed in hegemonic (i.e., white middle-class American) motherhood. In contrast to the white, middle-class American, gender ideology, Collins’s concept of mothering for women of color assumes the necessity of work outside the home; recognizes reliance on “other mothers” (i.e., beyond biological relations); and values the labor inherent in mothering. O’Reilly (2020) presented motherwork during the COVID-19 pandemic as frontline essential work.

Collins and fellow Black Feminists echoed other scholars of gender and noted that that normative motherhood (a) is also coded as white (b) assumes that all women are destined to be mothers, (c) that pregnancy and birth are physically seamless and easy processes for all women, and (d) every woman finds her ultimate purpose through mothering. With regards to parenthood, normative motherhood can translate into what Hays (1996) called intensive mothering, or the social expectation that mothers are constantly engaged in researching, perfecting, and engaging with child development. Arguably, this social demand for intensive mothering increased for all mothers during the COVID-19 pandemic, which we argue, acutely impacted Indigenous mothers. Many scholars of gender have noted the guilt, stress, blame, and social sanctions mothers can internalize when they are told or perceive that they are falling short of these normative expectations (Jackson & Mannix, 2004; Reiheld, 2015; Warner, 2005). We sought to highlight the resilience and transcendence that Indigenous motherwork fostered alongside the stress of normative mothering that women experience much more than fathers (or indeed, all men).

## Indigenous Motherwork

Mendoza (2023) attributed the legacy of slavery in the United States and persistent racism as significant drivers influencing Black maternal practices. Similarly, Mendoza (2023) connected the history of settler–colonial oppression and patriarchy to the current maternal practices and ideologies of Indigenous mothering, emphasizing the need to combat such insidious sources of oppression. Snitow (2015) underscored the impact of enduring systemic racism as creating a social and economic environment of crisis for Indigenous mothers; thus, Indigenous motherwork emphasizes the daily struggle for survival and community solidarity. Despite extensive scholarship on historical oppression and Indigenous resilience, little research has examined how these themes emerged during the COVID-19 pandemic, particularly in their impact on Indigenous women. The pandemic magnified pre-existing disparities, placing Indigenous mothers at the intersection of heightened caregiving responsibilities, economic precarity, and the struggle for healthcare accessibility. Examining Indigenous motherwork during this crisis highlights the resilience and adaptability of Indigenous maternal practices and labor in the face of systemic challenges.

## Framework of Historical Oppression, Resilience, and Transcendence

Alongside previous research on Indigenous motherwork, we used Burnette and Figley’s (2017) framework of historical oppression, resilience, and transcendence (FHORT) as a culturally congruent framework to understand the COVID-19 pandemic’s impact on Indigenous mothering and, likewise, Indigenous mothering’s impact on the unfolding conditions of the COVID-19 pandemic. Within this relational framework, historical oppression encompassed enduring, intergenerational, and chronic forms of oppression that have become internalized, imposed, and normalized. As a result of these compounded oppressions, Indigenous mothers have experienced a range of social and health disparities (Dalla & Gamble, 2001; Gurr, 2014; Liddell et al., 2021; McKinley et al., 2019). Historical oppression (e.g., colonialism, racism, capitalism, patriarchy) creates a foundation for an undue burden of responsibility placed on Indigenous women, despite their demonstrated resilience and capacity to transcend adversity through the interconnections of promotive and protective factors and risk across cultural community, individual, familial, and societal levels (Burnette & Figley, 2017).

We defined *resilience* as the capacity to recover from adversity and adapt and acquire skills to transcend oppression, shaped by context and dynamic and fluid (Masten & Monn, 2015; Waller, 2001). The interplay of risk and protective factors across various ecological levels determines whether an individual can experience resilience, transcendence, and overall wellness—encompassing physical, mental, emotional, and spiritual health—after facing adversity (Burnette & Figley, 2017). Moreover, we understood the formalized and informalized labor done by Indigenous mothers during the pandemic to sustain their families as a unique form of motherwork that creates sustenance and offers resilience to future generations within the FHORT.

### Reproductive Justice

Reproductive justice was an integral aspect of this study, as it places reproduction within the broader social context of racial and gender-based domination and oppression. We used this framework to shift the focus away from individual choice and instead underscore the significance of the resources available to women for making informed reproductive and parenting decisions. Moreover, as emphasized by Roberts (1991), enduring government control over the reproduction of women of color has targeted their reproductive autonomy and limited reproductive choices relative to white women who have racial privilege. The social justice lens acknowledges the historical oppressions that remain relevant today and confirms that government policies have historically exploited and targeted women’s bodies as a means of oppressing their own communities (Ross & Solinger, 2017). As Ross and Solinger (2017 articulated, the three core principles of reproductive justice encompass: “1. The right not to have a child, 2. The right to have a child. 3. The right to raise children in safe and healthy environments” (p. 9). Although the first principle has been a central aspect of white women’s concerns due to their racial privilege, women of color have focused on the right to have a child and raise them in a safe and healthy environment due to historical oppression.

Colonization stripped Indigenous women of institutional power and deliberately perpetrated ethnic genocide. Indigenous women’s reproductive capacities were targeted and employed as tools to assert white settler colonial power, accumulate wealth, and stake claims to Indigenous property (Baskin, 2020). Reproductive power was especially salient for matrilineal tribes, where power and wealth were passed down through the mother. Policies targeting Indigenous women’s power, such as the Indian Act of 1876, were vital in this context. The dehumanization of Indigenous women was central to the success of colonization (Baskin, 2020). Indigenous women’s reproductive rights were violated through practices such as forced sterilization, the removal of Indigenous children from their families for placement in boarding schools, and the criminalization of Native cultural practices (Gurr, 2014; Theobald, 2019).

Contemporary repercussions of colonialism and assaults on Indigenous women’s right to mother are exemplified by the disproportionate presence of Indigenous children in the foster care system (Lawler et al., 2012). The social justice framework provides essential insights into the erosion of the fundamental right to mother one’s children in Indigenous communities. Despite the targeted campaigns and horrifying atrocities inflicted upon Indigenous communities, particularly Indigenous women’s bodies, Indigenous mothers have persisted and ensured the continuity of their communities and asserted their right to mother. This study explored Indigenous motherwork as a practice of survival, resilience, and responsibility during the challenges and resource restrictions during a contentious COVID-19 pandemic environment.

### COVID-19 and Indigenous Communities

COVID-19 has disproportionately impacted Indigenous communities due to cultural suppression, ongoing settler–colonial oppression, and entrenched socioeconomic disadvantages (Power et al., 2020). The pandemic underscored and reinforced preexisting physical, social, and mental health disparities (Emerson & Montoya, 2021). Indigenous communities have faced longstanding challenges in healthcare access, economic opportunities, and education, contributing to their heightened susceptibility to COVID-19 and increasing adverse outcomes (Yellowhorse, 2022). Alongside Indigenous individuals facing hospitalization rates four times higher than the national average and significant reductions in life expectancy at birth (Goldman & Andrasfay, 2022; Indian Health Service, 2022; McKinley et al., 2023, structural inequities such as limited access to essential information, multigenerational living arrangements, and employment in frontline roles have further exacerbated their viral risk (Leggat-Barr et al., 2021; Rodriguez-Lonebear et al., 2020). These staggering inequities are linked inextricably to centuries of colonial oppression and the intergenerational trauma of Indigenous people (Burnette & Figley, 2017). Despite such challenges, Indigenous communities and individuals have exhibited remarkable resilience, embodying “survivance” as they continue to thrive in their culture, identity, and values (Baldwin et al., 2023).

### Indigenous Mothers

The disproportional weight of family responsibilities carried by U.S. mothers can lead to detrimental physical and mental health repercussions, including depression and cardiovascular diseases (Ciciolla & Luthar, 2019; Henderson et al., 2016; Manuel et al., 2012). Indigenous mothers are often the leaders of Indigenous families and communities (McKinley, 2023a, b; McKinley et al., 2021). Studies examining Indigenous mothers have further underscored the stark disparities and outcomes they face, such as an elevated risk of maternal morbidity and mortality (Gurr, 2014; Theobald, 2019), postpartum depression (Baker et al., 2005), and tragic instances of suicide (Curtin & Hedegaard, 2019).

In precolonial times, Indigenous maternal practices and knowledge were understood as pivotal cultural cornerstones, with women esteemed as essential tribal members and leaders (Brant, 2014, 2017). The interconnections of the natural world and ecological systems mirrored a holistic “family” perspective. Indigenous practices of mothering are intergenerational and expansive, with kinship networks extending to the wider community (Burnette, 2018; Burnette et al., 2020; McKinley, 2023a, b; Tam et al., 2017). Unlike the conventional settler–colonial distinction between public and private spheres as static social locations, Indigenous worldviews see these spheres as fluid and interconnected. Moreover, Indigenous motherwork is not confined to human relations but extends to include responsibilities toward the earth, land, water, and the spiritual realm (Brant, 2014, 2017; Udel, 2001). Indigenous spiritual beliefs centralize the planet as a living entity, highlighting the interdependence of all life forms and the value of feminine nurturing attributed to the earth. “Mother Earth” underscores the centrality of motherwork as foundational to Indigenous culture and rooted in spiritual cultural beliefs. Furthermore, Indigenous parental relations are not hierarchical like the settler–colonial patriarchal system. Takševa (2017) explained, “Indigenous motherhood involves understanding the reciprocal relationship existing between mothers and children, which honors the subjectivity of both mother and child and contrasts with patriarchal and oppressive ideologies of mothering” (p. 158). This egalitarian relational structure promotes autonomy, interdependence, and greater cohesion as a kinship.

In the Indigenous paradigm, motherwork is imbued with symbiotic, interdependent care systems, fostering responsibilities toward all living beings (Mendoza, 2023). Maternal practices are modeled after “native traditions of responsibilities.. to a personified, feminized earth” (Udel, 2001, p. 44). Indigenous motherwork places value on women’s ability to procreate and nurture not just their children and communities, but also the earth itself. These cultural values of responsibilities of care and the interconnectedness of mothering with the earth and broader systems of communal care set Indigenous mothering apart from settler mothering, which tends to focus on individual rights in relation to men (Udel, 2001). Indigenous cultural values and the centrality of mothering work in harmony with the broader interconnection of social and ecological systems. Indigenous motherwork “valorizes [women’s] ability to procreate and nurture their children, communities, and the earth” (Udel, 2001, p. 43).

### Single-Mother Households

Families led by single mothers who are low income represent a particularly vulnerable family type, especially in the context of the COVID-19 pandemic, with school closures having significant impacts (Radey et al., 2021). As of 2020, approximately 21% of U.S. children under 18 lived with their mother only (Hemez & Washington, 2021). Discrimination, safety concerns, and a lack of social and financial resources have long been recognized as burdens single-mother households face (Edin & Lein, 1997). Alarmingly, two thirds of families headed by single mothers lived below 200% of the poverty line during this time frame (Lu et al., 2020), often holding jobs in sectors with less job security and flexibility, such as the service industry or essential worker roles (Bobrow, 2020).

Indigenous children under the age of 6 are 14% more likely to experience poverty compared to the general population, with a staggering 65% of them living in families on the brink of poverty—a rate 20% higher than the overall population (Jiang et al., 2017). Moreover, estimates have revealed approximately 66% of Indigenous births are to unmarried mothers, a sharp contrast to the 40% observed in the broader population (Porter et al., 2019). The compounded social vulnerabilities experienced by Indigenous families led by single mothers have been further exacerbated by the challenges posed by the COVID-19 pandemic. Despite such overwhelming burdens, these resilient mothers persevered in the face of daily uncertainty, fear, and a lack of resources as they continued to carry out the essential work of their families and community through motherwork.

## Method

### Research Design

This qualitative research study assessed the impact of the COVID-19 pandemic on a subset of Indigenous women in the southeastern region of the United States. This research was conducted as part of the National Institutes of Health Notice of Special Interest call for COVID-19 research and was part of a larger critical ethnography to inform development and testing for the Weaving Healthy Families program (McKinley & Theall, 2021). This study was approved by Tulane University’s Institutional Review Board and all participants provided informed consent before data collection.

A total of 31 qualitative interviews were conducted in a community-based setting as part of this research. Carspecken’s (1996) critical ethnographic approach was used, as it focuses on empowering participants’ voices to disseminate knowledge (Burnette et al., 2014; Carspecken, 1996; McKinley et al., 2019). Participants (*N* = 31) were recruited from a southeastern reservation of a federally recognized tribe in the United States with eight distinct communities. The tribe has an independent infrastructure, including social/family services, leadership, and law enforcement, with distinct COVID-19-related safety protocols and regulations. All potentially identifying information was removed from the results and will remain confidential in accordance with tribal agreements and the Toolkit for Ethical and Culturally Sensitive Research (Burnette et al., 2014; McKinley et al., 2019). Our research applies Burnette et al.’s (2014) *Toolkit for Ethical and Culturally Sensitive Research*, which provides a framework for conducting research in alignment with Indigenous worldviews, values, and priorities. The toolkit emphasizes relationality, respect, and reciprocity, ensuring that research is not extractive but mutually beneficial. It guides researchers in engaging Indigenous communities through meaningful collaboration, honoring Indigenous knowledge systems, and upholding ethical considerations such as informed consent, community ownership of data, and culturally responsive dissemination of findings.

Using a community-based critical ethnography approach, we center Indigenous voices, examine systemic inequities, and promote actionable change. This methodology aligns with Indigenous storytelling traditions, which value direct speech and relational knowledge-sharing. To honor these principles, we use participant quotes as theme titles, ensuring authenticity, respecting narrative sovereignty, and maintaining accountability to those who shared their experiences, rather than imposing external categorizations. This approach reinforces the ethical and culturally attuned framework guiding our study and ensures that findings remain rooted in the perspectives of those most impacted by the research.

### Data Collection

Before initiating the research, the third author secured approval from the sponsor university’s institutional review board and the focal tribal council, along with written informed consent from all participants. The PI had developed long-term relationships with tribal members, who served as liaisons during study recruitment (Burnette et al., 2014; McKinley et al., 2019). Participants were asked to choose their interviewer, with 24 participants interviewed by the PI (77.42%) and seven by a tribal research team member (22.58%). Individual interviews were conducted in a conference room at a local facility between December 2021 and April 2022, and interview lengths ranged from 23 to 85 minutes (*M* = 51.42 minutes, *SD* = 14.12).

To semi-structure the conversation with participants, the PI developed an interview guide along with a tribal research team member (Burnette et al., 2014; Carspecken, 1996; McKinley et al., 2019). The guide, adapted to a fifth-grade reading level, addressed topics related to physical, mental, and spiritual changes among individuals and families and social changes in the broader community. Examples of relevant questions included: (a) How were you affected by the COVID-19 pandemic?; (b) What have you learned about yourself through the COVID-19 pandemic?; (c) How has the pandemic affected: your mental health, your parenting, your partner relationships, your priorities or values, your sense of meaning, spirituality, or life satisfaction?; and (d) How have you coped?

In all, 31 adult female tribal members who identified as head-of-household participants were interviewed. For time and participation, participants were paid $50 USD via Clincard, a reloadable debit card used in the Weaving Healthy Families program. Participants ranged in age from 29 to 56 years old (*M* = 37.97, *SD* = 6.34). All participants were in caregiving roles, and all but one participant was a mother (*n* = 30, 96.77%). Participants reported their relationship status, including single (*n* = 13, 41.94%); married (*n* = 11, 35.48%); in a relationship, but not married (*n* = 6, 19.35%); and widowed (*n* = 1, 3.23%). Household size ranged from three to nine (*M* = 5.61, *SD* = 1.96), and the number of biological children ranged from zero to eight (*M* = 3.55, *SD* = 2.16). For more demographic information, see Table 1.

**Table 1:** Participant Demographics

Variables	*n*, %
Age (range 29–56)	39.79 (*SD* = 6.34)
Community of residence	
*Main community*	15 (48.39%)
*Other communities*	16 (51.61%)
Highest level of education	
*Some high school*	4 (12.90%)
*High school graduate or equivalent*	4 (12.90%)
*Some college*	7 (22.58%)
*Associate degree*	7 (22.58%)
*Bachelor’s degree*	5 (16.13%)
*Master’s degree*	4 (12.90%)
Employment	
*No employment*	5 (16.13%)
*Part-time employment*	1 (3.23%)
*Full-time employment*	25 (80.65%)
Household size (range 3–9)	5.61 (*SD* = 1.96)
Number of biological children (range 0–8)	3.55 (*SD* = 2.16)

*Note*. (*N* = 31)

### Researchers Positionality

Our research team acknowledges the critical importance of cultural positionality in shaping our engagement with Indigenous communities. We understand that our diverse backgrounds, perspectives, and potential biases can influence various aspects of the research process, including data collection, analysis, and interpretation. Within our authorship team, two members identify as Indigenous, and our broader team includes Indigenous individuals who have contributed in various capacities, such as community health representatives, community advisory board members, and consultants. Additionally, tribal community members were invited to participate in reviewing and co-authoring the manuscripts, enhancing the authenticity and cultural relevance of our findings.

For team members who do not identify as Indigenous, we have taken intentional steps to practice cultural humility through ongoing reflection, dialogue, and collaboration with Indigenous partners. These efforts are aimed at ensuring our research is conducted respectfully and aligns with the priorities, values, and knowledge systems of the Indigenous communities involved. Each team member brings unique cultural backgrounds, experiences, and roles within the broader Community-Based Participatory Research (CBPR) framework that informs this study.

The first author is a PhD student who is a dedicated ally and committed to supporting Indigenous-led research and amplifying community voices through ethical, collaborative scholarship. The second author is an academic ally who contributes her skills to support community-driven goals through a culturally responsive and collaborative approach. The third author is the Principal Investigator for Weaving Healthy Families and a long-term ally who has collaborated with the tribe for over a decade. Her work emphasizes building and maintaining partnerships grounded in trust, mutual respect, and shared goals. The fourth author is an Oklahoma Choctaw citizen and researcher who focuses on advancing Indigenous health through culturally grounded methodologies that center Indigenous knowledge systems and healing practices. The last author is a Native Hawaiian and Program Manager with Weaving Healthy Families and contributes invaluable lived experience and cultural insight into Indigenous family well-being and resilience.

Our collective positionality reflects a blend of Indigenous and non-Indigenous perspectives, united by a shared commitment to ethical, culturally responsive, and community-centered research. We acknowledge the historical and ongoing impacts of colonization and strive to ensure that our work is conducted with humility, respect, and reciprocity. Central to our approach is prioritizing the inclusion of Indigenous voices and leadership, recognizing that the knowledge, lived experiences, and cultural wisdom of Indigenous peoples are integral to the research process.

### Data Analysis

The research team included trained analysts from diverse backgrounds, including tribal and nontribal students at master, doctoral, and postdoctoral levels and Indigenous informants with experience with the focal tribe (McKinley et al., 2019). Interviews were audio recorded by the PI and transcribed verbatim by a professional transcription service, then reviewed for accuracy and holistic immersion by multiple research team members. The first and fifth author then coded several interview transcripts in NVivo, a qualitative analysis software, as examples and utilized thematic analysis. This hierarchy was adapted as more interviews were coded. The third author held multiple follow-up meetings over the months-long review process to report progress and engage with preliminary discoveries. Manuscripts were then drafted in an incremental approach that incorporated and synthesized feedback from the PI.

### Data Trustworthiness

Ensuring data trustworthiness is central to the rigor and reliability of this study. To establish trustworthiness, we adhered to the four widely recognized criteria for qualitative research: credibility, transferability, dependability, and confirmability (Lincoln & Guba, 1985, p. 290). To ensure credibility, or confidence in the truth of the findings, we employed multiple strategies, including prolonged engagement, triangulation, and member checks. The third author developed sustained long-term relationships with tribal members prior to the research process, fostering trust and familiarity. Triangulation was achieved through multiple data sources, including interviews with community health representatives, community advisory board members, and consultants. Additionally, member checks were conducted by providing participants with copies of their transcripts to review and revise, ensuring that their experiences were accurately captured and reflected. A detailed summary of all themes and quotes was created and shared with participants, cultural insiders, and the focal tribe.

To enhance transferability, or the applicability of findings to other contexts, we provided rich, thick descriptions of the study’s context, participants, and procedures. Detailed demographic information was collected and reported, including participants’ age, relationship status, household size, and caregiving roles. By offering comprehensive contextual details, we allow readers to assess the applicability of our findings to other Indigenous communities and settings. Dependability, or the consistency and repeatability of findings, was addressed through a meticulous coding process, ongoing peer debriefing, and documentation of methodological decisions. The third and fifth authors created a preliminary hierarchy of themes using NVivo software, which was adapted as more interviews were coded. An audit trail of coding decisions, changes, and reflections was maintained throughout the research process, ensuring transparency and consistency.

To achieve confirmability, or neutrality in findings free from researcher bias, we incorporated strategies such as reflexivity, collaboration, and external audits. Team members, including both Indigenous and non-Indigenous researchers, engaged in continuous reflection and dialogue to mitigate bias and ensure findings were shaped by participant perspectives rather than researcher assumptions. Furthermore, collaboration with Indigenous partners and community members throughout data analysis and manuscript development contributed to the credibility and authenticity of the findings. Collectively, these measures ensure that our research maintains a high standard of rigor, authenticity, and cultural responsiveness. The strategies outlined above align with the frameworks guiding our study, promoting a process that is ethically sound, culturally attuned, and rooted in the lived experiences of Indigenous women.

## Results

This study focused on analyzing findings pertaining to the specific theme, *How Affected by COVID-19: Motherwork*, from the data. The motherwork undertaken by women of color, as described by Collins (1997), is deeply rooted in distinct racialized frameworks encompassing survival, power, and identity. Mothers’ pursuit of each of these elements is intricately interwoven with their encounters with systemic oppression and their aspiration to secure a future where their children can thrive despite the challenges posed by a racially discriminatory society.

Using this framework, several overarching themes of motherwork emerged during the qualitative analysis: (a) *Prioritizing Survival Need: “more survival skills.. you put a lot of stuff to the back corner*”; (b) *Navigating Oppressive Forces: “not my fault but.. they might put me in jail”*; and (c) *Embracing Caregiving as both Labor and Leadership: “this is on me.”* These themes illustrate the fluid and porous boundaries of motherwork, highlighting its impacts intricately intertwined with their children’s well-being, extended kinship networks, and the wider community. The emerging themes and descriptions and the frequency of occurrence are summarized in Table 2.

**Table 2:** Summary of Main Themes of Motherwork

Theme	Description	Example	Frequency
Prioritizing Survival Need	Indigenous mothers prioritize survival needs to nurture their children and communities amid systemic oppression. Survival is rooted in relationality and the awareness of colonizer devaluation.	“More survival skills.. You put a lot of stuff to the back corner. You know, and that was a part that I hated to teach my daughter.. Don’t worry about you right now. We’re going to have to do this right now.”	18
Navigating Oppressive Forces	Indigenous mothers navigate systemic oppression, including colonialism, patriarchy, and sexism. They exhibit resilience through humor, empathy, adaptability, and interdependence.	“[I said], I’m just going to have to send y’all to school. Because, this truancy.. [The school and law enforcement] don’t play.. They might put me in jail.”	10
Embracing Caregiving as both Labor and Leadership	Indigenous motherwork encompasses both labor and leadership. It involves nurturing and providing for families, kin, and Indigenous culture, despite increased responsibilities and expectations.	“[My children] shouldn’t be doing this, ‘cause [sic] this is on me.”	14

### Prioritizing Survival Needs: “More Survival Skills... You Put a Lot of Stuff to the Back Corner”

Survival for Indigenous mothers goes beyond safety measures to nurture the well-being of their children and communities. Indigenous survival is rooted in the awareness of colonizer devaluation of Indigenous worth and contributions, along with the disproportionate penalization within justice systems (Baskin, 2020). Indigenous motherwork is deeply rooted in the notions of interconnection and relationality. Throughout interviews on the impact of COVID-19, most participants focused on the well-being of others, particularly their children. One participant (41 years-old, married, three children, not working outside the home) vividly captured the symbiotic relationship between mothers and their children amid the strains of the pandemic, stating, “Everything I was going through affected [my son]... Something’s gotta [sic.] give.” This mother articulated the reciprocal relationship between her child and herself, acknowledging her stress during the pandemic was directly impacting her son and recognizing the urgent need for relief.

Another related cornerstone of motherwork is survival and prioritizing essential necessities. Many participants discussed the necessity of prioritizing fundamental survival needs during the pandemic and having to push aside nonurgent needs and desires. A central theme revolved around prioritizing basic survival needs during the pandemic, often necessitating the postponement of nonurgent desires and wants. Kellie (36 years-old, living with partner, seven children, not working outside the home) expressed remorse about teaching her daughter this kind of survival skill. She said:

More survival skills.. You put a lot of stuff to the back corner. You know, and that was a part that I hated to teach my daughter.. Don’t worry about you right now. We’re going to have to do this right now.

The pandemic disrupted the caretaking of children and domestic duties largely through school not being readily accessible to children. Such a change shifted participants’ priorities to focus exclusively on the most urgent needs. Pamela (41 years-old, married, three children, working full-time outside the home) commented on the inconsistent availability of schooling for her children and the tireless impacts. She shared:

This time they didn’t go to school for about 2 weeks.. We couldn’t catch a break. One of them gets sick, then we all had to quarantine. Then one gets healed, another one gets sick. It was about a whole month. I enrolled them, they didn’t go to school.

Another participant Genevieve (37 years-old, single, living with extended family, one child, working full-time outside the home) echoed how she had a survivalist mentality in trying to work and mother while her children did school remotely at home. She expressed distress in not knowing how to meet all the demands of her family and work during the pandemic and said:

When I get off [work] after 3:00 AM, I would sleep through [my alarm] and the kids would miss school constantly. I wouldn’t get up till like 2:00 [P]M and the [online schooling start time] was like at 9:00, 10:00 in the morning. So, it’s like I said, “I don’t know what I’m gonna [sic.] do.” They were failing even though they knew how to do it.

Many participants discussed the added motherwork and additional responsibilities of remote schooling. Janie (29 years-old, married, one child, working full-time outside the home) expressing fatigue said, “I hope it’s over this year ’cause [sic.] I’m ready for them to go back to school.” Another mother, Sheryl (43 years-old, married. four children, working full time outside the home) discussed how technology failures resulted in punitive legal action for her and her child and noted:

When he started virtual because, his computer had a breakdown, or it wiped out all his stuff. So, he had to redo some of the [work]. [The school] considered that [as] him being absent. So, I had to go to court twice.

Another participant Margaret (38 years-old, single, five children, working full-time outside the home) expressed concern about her child’s developmental and behavioral progress during the pandemic due to the unavailability of early childcare, stating:

My daughter’s been affected with us being quarantined when she was still a baby and in that transition phase. Now we’re having problems with her. She doesn’t speak, and she’s 3. She has behavioral problems. She doesn’t do well in public. We’re having to do speech and occupational therapy.

This mother expressed her consuming worry about her young daughter falling behind developmentally due to a lack of social and educational resources available to her.

Lack of schooling outside the house and childcare increased participants’ physical and emotional loads. The pandemic expanded participants’ motherwork load by charging them with being health protectors from the COVID virus. Participants had to “learn more boundaries” and enforce safety precautions with their families. One mother Marion (43 years-old, single, three children, working part-time outside the home) shared:

We had to be very careful. I had to be on them [sic.], “Keep your hands clean, wash your hands, use sanitizer.” Especially the masks, I have to enforce the mask. Anytime you go anywhere, we always had to have it.

Most mothers expressed a constant concern for the health of their family and kin and themselves and tireless efforts at keeping their family safe through enforcing safety protocols and restricting in-person social interactions for their households. Still, some participants expressed positive results from new boundaries, such as better communication and openness among their families. Lela (40 years-old, single, five children, working full-time outside the home) said:

[I want] them to be open with me, if something was bothering them, [I want] them to.. just be open with me and tell me things so we can talk about it, try to fix it if it can be fixed.

School closures and lack of available childcare directly impacted most participants’ ability to work and, subsequently, financial situations. One mother, Monique (36 years-old, married, six children, working full time outside the home) expressed this direct relationship, saying:

I have missed so much work since 2020. It’s been bad. I feel like I can’t even catch up no more [sic.] and go into work on time no more [sic.]. I usually get there at 9 o’clock every day [before the pandemic]. [Missing work] is affecting us a lot money-wise.

The lack of childcare from schools, not being physically open directly impacted many mothers’ ability to perform job duties as they had previously. In many cases, this meant reduced or modified work schedules, which decreased their income. This change in employment was especially salient in families with single mothers as sole financial providers for their children. Lela discussed the impacts of uncertainty of the children’s school modifying her work schedule and shared:

With work [and] being a single parent.. adjusting to school closing early.. and me being the program manager here.. we have to modify our schedules and we’ve just got to work with.. what Chief’s orders are.

Single-mother households require a high level of adaptability and flexibility to meet their obligations as parents and providers for their children.

These mothers expressed how the pandemic heightened their emotions. The additional motherwork of schooling children from home while working, financial stress, and being virus protectors contributed to the emotional and mental stress of mothers and the children in their care—specifically the fear of death from COVID-19. Marion described her child’s fear of her death, saying, “[It] put a fear in her, thinking, ‘if I catch it, am I gonna [sic.] die?’” Another mother described how the prevalence of death in the community was impacting her children and herself. She stated, “I think it was more the sadness.. the death and the death rate. That made us feel depressed.. knowing that sadness was around.”

Fear of illness and death was a distinct and persistent element of motherwork as survival during the pandemic for participants. This theme was especially salient for families with one parent on whom the family was dependent, Marion described:

I don’t [want] nothing [sic.] to happen to me.. because I’m the single parent. I’m raising all these kids. My kids had that fear, too, I hope it’s not [my mother], I hope this doesn’t happen to us.

### Navigating Oppressive Forces: “Not My Fault But... They Might Put Me in Jail”

Participants discussed continuing to grapple with the enduring influence of oppressive social structures such as colonialism, patriarchy, and sexism, further compounding the challenges of motherwork during the pandemic through restrictions of resources and social networks. In this context, power was defined as the extent to which the mothers had control over their lives and how they navigated, adapted, and resisted these forces. Indigenous motherwork encompasses a commitment to navigating systemic obstacles and ensuring their children’s success despite pervasive discriminatory practices. The pursuit of power and control is imperative for Indigenous mothers, as it is a means of equipping their families with the tools to confront and transcend systemic barriers (Baskin, 2020; Udel, 2001).

Sheryl described the criminal implications she could face for her children’s truancy. She explained to her children the lack of control available to her when facing governmental power despite not being at fault. This mother used humor to deal with systemic oppression and deliver a hard reality to her children while modeling a positive coping practice for them to emulate. She recalled:

[I said], I’m just going to have to send y’all to school. Because, this truancy.. [The school and law enforcement] don’t play. It’s not your fault, it’s not my fault. But, at the same time, if you keep missing your work, they might put me in jail. I ain’t got time [sic.] to be sitting in jail. [laughed].

Another participant Luz (46 years-old, single, three children, working full-time outside the home), shared her experience of losing her husband and the simultaneous loss of traditional cultural practices because of pandemic-related restrictions. She described a lack of autonomy during a time of great vulnerability and said:

My husband died of COVID-19. [During that time], we were all positive [for COVID-19]. My oldest granddaughter, she was at her mother’s house. So, it took maybe almost a month before we buried [my husband].. Not being able to bring him home, we were not able to do what our traditional way was.

She explained the difficulty of losing her husband and vital cultural practices simultaneously.

Motherwork was also expressed as a site of resistance to oppressive forces and resilience in maintaining cultural ways and indigeneity. Sheryl articulated the collectivist value of teamwork and togetherness and said, “I’ll tell her we’re a team. And we can do this together.. Everything that I do and say is what I’ve learned in the in the past.” Interdependence and previous knowledge in overcoming obstacles were identified as ways to transcend challenges.

Victoria (35 years-old, single, three children, working full-time outside the home) expressed her unified community as a place of support for her raising her son. She explained the connection and support from other community members in caring for and watching her son and shared:

Red Water is just one community. You go and do something, you get [in] trouble somewhere.. somebody in this community is gonna [sic.] see you and it will get back to me or is going to get back to your daddy. I said.. get in front of [it], let us know what happened, tell us the truth, and then we’ll deal with it from there.

Marion underscored the value of family unity and togetherness, and stated, “But it also brought us together.. as a family we did more things. At home, we got to work on different projects.” This mother described using the social isolation of the pandemic to promote family cohesion and teamwork.

Participants expressed support of kin (e.g., family, friends), employers, community, and resources as instrumental to motherwork. One woman said her boss’s allowances for her childcare responsibilities helped her greatly. The support of other women was also detailed as being instrumental in dealing with daily challenges. One mother described, (Marion) “I had a large support group and my mother had her support group and.. and we talked about it. That was the thing.” Communal care and support were identified as vital elements of motherwork, which helped mothers exercise the power and control available to them.

Genevieve realized her community needed access to healthcare and resources: “It made me more aware of things that our tribe needs better health care, how can we do this to help our people?” This participant identified the structural shortcomings and lack of resources as the primary driver of problems facing her community due to the pandemic. Supportive traditional cultural practices were discussed as instrumental but limited by social-distancing protocols, especially regarding wakes and funerals. Marion described how not having time to be with her loved one’s deceased body and be with kin left her feeling rushed and unable to accept the death. She said:

The process was very different because the traditional way of having a funeral service.. you get to see and sit with the body, we couldn’t do a wake, there was no families coming together to mourn.. Move on to the next, move on to the next.. My kids’ grieving process was different, they felt rushed, and it was like they.. didn’t know how to accept it.

This mother articulated the impact of structural forces on essential social and cultural practices, shaping individual and communal experiences of grief and acceptance of death profoundly.

The concept of motherwork, as articulated by these women, illuminated the complexities and contradictions inherent in navigating social distancing measures from the COVID-19 pandemic. These practices, although aimed at ensuring physical safety, often come at the cost of individual and communal social, spiritual, and emotional well-being and health. Social-distancing protocols that prioritize individual physical safety might align more with individualistic cultures and values, such as white settler–colonial ideals, potentially impacting communalist Indigenous cultures in distinct and more significant ways. Despite facing structural limitations, many of these mothers exhibited remarkable resilience, employing humor, empathy, and adaptability while nurturing and supporting others. In doing so, they reclaim the power available to them within these constraints.

### Embracing Caregiving as Both Labor and Leadership: “This Is on Me”

Dutiful care in the context of motherwork refers to mothers’ conscientious and responsible care, often driven by a sense of duty and commitment to their identity as caregivers. This care is linked intrinsically with maternal identity and commitment as caregivers; nurtures; and providers of families, kin, and Indigenous culture. In the broader framework of motherwork, this term embodies the care, support, and nurturing that mothers provide for their children and families, encompassing the physical and emotional aspects of their responsibilities.

Many mothers expressed guilt when their children helped with duties they considered as their own. One mother Jenna (47 years-old, single, two children, working full-time outside the house) articulated, “[My children] shouldn’t be doing this, ‘cause [sic.] this is on me.” Despite the extensive responsibilities involved in motherwork, plus the additional load brought on by the pandemic, expectations increased, and many mothers expressed these expectations as solitary burdens for them to bear. Michelle (48 years-old, single, four children, not working outside the home) explained the importance of self-reliance as essential to the identity of motherwork; she described what her father taught her about parenting, stating, “Don’t rely on anybody. Don’t expect handouts. If you have kids, you have to take care of them, regardless of whatever.” This statement underscored self-reliance and care as essential to motherwork regardless of external circumstances.

The individual expectations and expansive responsibility of motherwork did not waver despite the challenges or increased load from the pandemic. Jenna described her guilt from her children helping her despite her diminished capacity and ceaseless responsibility of care. She shared:

[I] started cooking dinner.. I guess being so tired, when I sit down on the chair, I just fall asleep. [Then my daughter’s] up finishing it. She’ll literally finish dinner. She’ll give the two 1-year-olds and the two 5-year-olds a bath. She makes sure the 11-year-old takes a shower and [she’ll get] them all in bed, and Mariela’s probably going to bed about 12:00 or 1:00 in the morning. I said, “When I fall asleep like that, wake me up. Accept the fact that you have school yourself. You have your own schoolwork. I get it, I’m tired. But still.”

Despite this mother’s physical need for sleep, she said she desired to meet her motherwork responsibilities and expectations.

Many mothers reflected on their mothering identities in relation to their parents and their own experiences of being parented, revealing the cultural importance of generational ties. Kellie said her experience of being parented had her want to parent differently. She stated, “I tried to be different from [the way] my parents raised [me].. breaking that cycle.” Other mothers reflected on their children replicating their parenting and behaviors. Most mothers described how their motherwork and identity as mothers were impacted by their parents and experiences with care growing up.

Allison (29 years-old, not married, three children, working full-time outside the home) reflected on her daughter’s actions being like hers toward her mother when she was a similar age. She identified and felt compassion for her mother: “It’s like me looking at myself.. putting me through what I put my mom through.” Another participant Muriel (41 years-old, single, four children, working full-time outside the home) reflected a similar sentiment with appreciation: “Some days I sit back and think, gosh, my mom did all of this, she had to carry this weight.” She illuminated how this strengthens her and serves as a model for her motherwork in challenging times.

Some participants expressed taking time for themselves to cope with the unrelenting responsibilities of motherwork during the global pandemic. One participant Joan (34 years -old, married, four children, working full-time outside the home) expressed, “Sometimes need a little breather so you can get that tank filled up a little bit and get the energy back.” She described caring for herself as a necessary aspect of motherwork that restored her, providing energy to return to care and motherwork. She explained to her daughter how taking time away from motherwork is restorative and shared:

I tell my older daughter, when I say I want time to myself, sometimes it doesn’t mean I want the whole night or whole day to myself. It might just mean I need 2, 3 hours just to myself.. and then you all come back.

These mothers highlighted the importance of taking brief respites and prioritizing self-care as crucial components in fulfilling their motherwork duties and commitments. In doing so, they model the practice of self-nurturance for their children and exemplify the meaning of care.

## Discussion

This study highlights how Indigenous motherwork functioned as an essential site of survival, cultural preservation, and resistance during the COVID-19 pandemic. The findings illustrate how Indigenous mothers bore intensified caregiving burdens, balancing economic precarity, systemic oppression, and emotional labor while ensuring the survival and well-being of their families and communities. Three central themes emerged: (1) Prioritizing Survival Needs, in which mothers prioritized essential needs and relied on mutual aid networks; (2) Navigating Oppressive Forces, where participants contended with systemic exclusions, including childcare shortages, economic instability, and punitive legal surveillance; and (3) Embracing Caregiving as both Labor and Leadership, which demonstrated how mothers upheld intergenerational knowledge, resisted settler-colonial erasure, and reinforced community survival through caregiving.

### Indigenous Motherwork as Political Resistance and Cultural Resilience

These findings reveal that Indigenous motherwork extends beyond individualized maternal roles and operates as a site of political resistance, cultural sustenance, and collective care. Analyzing these mothers’ experiences through the lens of motherwork highlights the intricate labor, resilience, and communal strength embodied by Indigenous communities during the pandemic. Indigenous motherwork, as exemplified by these mothers, surpasses normative definitions of motherhood, showcasing motherwork as a potent means of communal resilience, autonomy, and cultural connection. Motherwork helps to explain the value these women provided to their families as frontline workers and community leaders in the overlapping domestic and public spheres. Throughout the pandemic, Indigenous mothers enacted strategies of adaptation and mutual support, ensuring the survival of their kin and reinforcing communal resilience amid multiple layers of oppression. Their experiences reflect broader patterns of Indigenous survivance, in which caregiving is deeply intertwined with decolonial resistance. Despite their critical role in mitigating the pandemic’s impact, Indigenous mothers’ labor remains unrecognized, mainly in dominant public health and policy discourses.

### Structural Barries and Systemic Inequalities

Participants faced severe constraints due to the intersection of economic precarity, legal restrictions, and inadequate access to culturally responsive resources. The pandemic exacerbated structural inequities, underscoring the urgent need for systemic reforms. Many Indigenous mothers struggled with school closures, which intensified caregiving demands while limiting economic opportunities. Additionally, the pandemic expanded participants’ motherwork load by charging them with being health protectors from the COVID-19 virus. Participants had to “learn more boundaries” and enforce safety precautions with their families, constantly navigating risks while ensuring their households’ well-being. Government policies, such as truancy laws, disproportionately impacted Indigenous families, reinforcing state control over Indigenous mothering. These structural inequities demonstrate how colonial systems continue to regulate and undermine Indigenous motherwork, restricting autonomy over caregiving practices.

### Interdependence and Community Support

Across the findings, motherwork emerged as the relational care interaction between a mother, her children, and their community. The nature of this motherwork was iterative and heavily reliant on the resources available to her—be it health, emotional well-being, or financial stability. It was steered by a culturally derived maternal identity rooted in an intrinsic sense of responsibility for both kin and community. However, the immense burden of motherwork—particularly for single mothers—often resulted in exhaustion and emotional distress. One mother vividly described this strain, recounting how she instructed her children to wake her up despite her physical exhaustion instead of assisting with her responsibilities. She felt the sole weight for the motherwork and expressed, “I get it, I am tired. But still.” The absence of support and resources, coupled with escalating expectations in physical and emotional motherwork, could escalate burnout and have enduring negative consequences if not addressed.

### Resistance and Cultural Survival Through Motherwork

Despite these challenges, this study highlights how motherwork is a form of resistance against settler-colonial structures. Indigenous mothers’ caregiving was not just a matter of necessity but an assertion of sovereignty, cultural survival, and political agency. Motherwork, seen as a form of activism and cultural revitalization (Brant, 2020), was acknowledged for its positive impact on community well-being and care recipients. However, this perspective can challenge mothers, as expectations and duties remain unchanged despite lacking resources or support. The accumulation of additional motherwork duties and responsibilities amid diminishing resources was often linked to descriptions of exhaustion and guilt among mothers who felt unable to meet the escalating demands of motherwork.

### Interconnectedness and Collective Support

Despite the enduring commitment and responsibility toward their kin, these American Indian mothers were acutely aware of their interconnectedness and dependence on others within the community for support and survival throughout the pandemic. Whether receiving assistance from “other mothers” (Collins, 1994) to navigate remote schooling or benefiting from accommodating employers who provided the necessary flexibility to engage in public and private motherwork, interdependence was a recurring theme. Community solidarity and support have long been recognized as fundamental aspects of Indigenous mothering, countering the impacts of colonial-settler oppression (Mendoza, 2023; Snitow, 2015), a sentiment consistently echoed by these women. One mother articulated the unity and support within her community, stating, “Redwater is just one community.” This discovery aligns with existing literature highlighting the health advantages of Indigenous cultural connections and solidarity (Walls et al., 2022) and the positive impact of strong family connections and relationships (Burnette et al., 2020). Through intergenerational knowledge transmission, Indigenous mothers preserved traditional mothering practices, reinforcing a relational and reciprocal model of care that challenges the individualistic, Eurocentric paradigm of motherhood. This aligns with broader feminist and Indigenous critiques of mainstream maternal theory, which often fails to account for the collective, decolonial nature of Indigenous caregiving.

The pandemic exacerbated entrenched disparities rooted in colonial oppression, particularly in healthcare and economic resources, profoundly affecting Indigenous communities (Connolly et al., 2021; Fortuna et al., 2020; Leggat-Barr et al., 2021). Many mothers described the implementation of social distancing and isolation protocols as a hindrance to crucial cultural practices, particularly in the context of funerals. This limitation had detrimental effects, preventing families from engaging in vital traditional expressions of grief, connection, and solidarity. It hindered motherwork and limited autonomy in caring for kin and community at a particularly vulnerable life transition; for instance, one mother expressed the inability to partake in traditional funeral practices for her deceased husband, deeming such a loss as “everything.”

These findings reinforce motherwork as a powerful form of survival, power, and identity for Indigenous mothers, families, and their communities during a public health crisis. As previous literature has demonstrated, Indigenous motherwork is a powerful form of activism in its various forms, addressing colonialism, sexism, and other forms of oppression and social injustices (McKinley, 2023a, b; Udel, 2001). While Indigenous women have historically endured the dual burdens of caregiving and systemic marginalization, their motherwork has persisted as a vital mechanism of cultural survivance. The experiences described in this study illustrate the necessity of understanding motherwork not as domestic labor alone but as an active political force shaping Indigenous resistance and resilience.

Analyzing these mothers’ experiences through the lens of motherwork highlights the intricate labor, resilience, and communal strength embodied by Indigenous communities during the pandemic. Indigenous motherwork, as exemplified by these mothers, surpasses normative definitions of motherhood, showcasing motherwork as a potent means of communal resilience, autonomy, and cultural connection. Motherwork helps to explain the value these women provided to their families as frontline workers and community leaders in the overlapping domestic and public spheres. These findings support motherwork as a powerful form of survival, power, and identity for Indigenous mothers, families, and their communities during a public health crisis. As previous literature has demonstrated, Indigenous motherwork is a powerful form of activism in its various forms, addressing colonialism, sexism, and other forms of oppression and social injustices (McKinley, 2023a, b; Udel, 2001). These mothers, with their unique position as southeastern American Indian tribal members, voiced their challenges and triumphs in motherwork throughout the pandemic.

Across the findings, motherwork emerged as the relational care interaction between a mother, her children, and their community. The nature of this motherwork was iterative and heavily reliant on the resources available to her—be it health, emotional well-being, or financial stability. It was steered by a culturally derived maternal identity rooted in an intrinsic sense of responsibility for both kin and community. Results highlighted self-reliance as a cornerstone of the motherwork identity, as expressed by a mother who emphasized, “This is on me.”

Motherwork, seen as a form of activism and cultural revitalization (Brant, 2020), was acknowledged for its positive impact on community well-being and the recipients of care. However, this perspective can pose challenges for mothers, as expectations and duties remain unchanged even when resources or supports are lacking. The accumulation of additional motherwork duties and responsibilities amid diminishing resources was often linked to descriptions of exhaustion and guilt among mothers who felt unable to meet the escalating demands of motherwork. One mother vividly described this strain, recounting how she instructed her children to wake her up despite her physical exhaustion instead of assisting with her responsibilities. She felt the sole weight for the motherwork and shared, “I get it, I’m tired. But still.” The absence of support and resources, coupled with escalating expectations in physical and emotional motherwork, could escalate burnout and have enduring negative consequences if not addressed.

Despite enduring commitment and responsibility toward their kin, these American Indian mothers were acutely aware of their interconnectedness and dependence on others within the community for support and survival throughout the pandemic. Whether receiving assistance from “other mothers” (Collins, 1994) to navigate remote schooling or benefiting from accommodating employers who provided necessary flexibility to engage in public and private motherwork, interdependence was a recurring theme. Community solidarity and support have long been recognized as fundamental aspects of Indigenous mothering, countering the impacts of colonial-settler oppression (Mendoza, 2023; Snitow, 2015), a sentiment consistently echoed by these women. One mother articulated the unity and support within her community, stating, “Redwater is just one community.” This discovery aligns with existing literature highlighting the health advantages of Indigenous cultural connections and solidarity (Walls et al., 2022) and the positive impact of strong family connections and relationships (Burnette et al., 2020).

### Cultural Continuity and De-colonizing Care

The pandemic exacerbated entrenched disparities rooted in colonial oppression, notably in healthcare and economic resources, profoundly affecting Indigenous communities (Connolly et al., 2021; Fortuna et al., 2020; Leggat-Barr et al., 2021). The FHORT contextualizes the historical and contemporary landscape of oppression experienced by American Indian women during the pandemic within colonial systems, particularly in the realm of motherwork. It sheds light on the backdrop shaping these women’s motherwork, merging their childhood experiences to acknowledge them as recipients and providers of care.

Many mothers expressed how their approach to motherwork was influenced by, or in reaction to, their parents’ efforts, aiming to break intergenerational cycles of trauma through their caregiving. They emphasized the reciprocity of support—whether it was receiving assistance such as sick days from a colleague, engaging in heartfelt conversations, or providing support by “just talking”—as essential to their motherwork, especially in overcoming the challenges posed by the pandemic. This mutual care exchange exemplifies Indigenous cultural cohesion (Masotti et al., 2023), vividly experienced through motherwork, nurturing social bonds, and fostering interdependence from oppressive forces.

Many women described the implementation of social distancing and isolation protocols as a hindrance to crucial cultural practices, particularly in the context of funerals. This limitation had detrimental effects, preventing families from engaging in vital traditional expressions of grief, connection, and solidarity. It hindered motherwork and limited autonomy in caring for kin and community at a particularly vulnerable life transition; for instance, one mother expressed the inability to partake in traditional funeral practices for her deceased husband, deeming such a loss as “everything.” These social distancing measures, reflecting the cultural values of individualism prevalent in settler–colonial U.S. society, overlooked the significance of collectivism within Indigenous cultures. Consequently, the cultural costs of social isolation may have had a greater impact on Indigenous communities reliant on social ties, communal care, and traditional practices.

### Reproductive Justice and Structural Inequalities

Reproductive justice frameworks shed light on the ongoing struggles against historical exploitation and the persistent denial of autonomy over reproductive choices faced by Indigenous women. A key aspect illuminated by these mothers is their quest for autonomy and the fundamental right to provide a safe and healthy environment for raising their children (Ross & Solinger, 2017). However, preexisting colonial structural inequities in healthcare access, socioeconomic disadvantages, and limited resources were further exacerbated during the pandemic, intensifying the challenges these mothers encountered.

Many mothers spoke of the significant financial constraints they faced while shouldering the primary—and often sole—caregiving responsibilities for their children, alongside managing professional roles and family finances. This struggle was particularly acute in single-mother Indigenous households, facing reduced social support and heightened socioeconomic vulnerabilities. As public schools shifted entirely to remote learning, mothers with young children were constrained to stay home, relying on limited kin support or seeking scarce and often expensive childcare services to sustain their careers and economic stability.

Adapting swiftly to the rapidly changing landscape of school closures and quarantines, these mothers demonstrated nimble adaptability. They harnessed motherwork and creativity to meet obligations within and outside the home, relying on support structures and care networks. Moreover, amid these challenges, results unveiled resilience and strengthened family cohesion, with mothers describing the experience as bringing them “closer together.”

The closure of physical schools significantly impacted these mothers’ lives, directly impacting their families’ financial stability and amplifying the mental and emotional strain of tending to children navigating the social and emotional challenges triggered by the COVID-19 global pandemic. Mothers highlighted the multifaceted repercussions of school closures on their children, from mental health struggles to hindered social development, adding to the already considerable burdens these mothers bore. Despite grappling with their own challenges when sharing their experiences, most mothers focused on their children and the issues affecting them, displaying great empathy. This finding aligns with the egalitarian structure of Indigenous parenting approaches and motherwork, which view children as autonomous subjective team members, emphasizing shared responsibilities and mutual consideration within the family unit (Takševa, 2018).

### Limitations and Future Research Directions

This study sought to enhance the understanding of how Indigenous motherwork was practiced during the COVID-19 pandemic, but certain limitations must be acknowledged. These insights are specific to the experiences of mothers from this tribe. Additionally, the retrospective nature of the inquiry raises a potential limitation. Participants were asked to reflect on their experiences during the COVID-19 global pandemic, which might not fully capture the dynamic nature of experiences during the pandemic nor the long-term implications. Additionally, this study highlighted the limited literature on Indigenous mothers in public health crises, emphasizing the need for more robust research that centers mothers and their unique practices and roles within their communities to inform effective interventions and support mechanisms.

Existing research remains fragmented, with Indigenous mothering often overlooked in maternal studies, public health discourse, and crisis response frameworks. This underscores the urgent need for Indigenous-led scholarship that foregrounds Indigenous maternal knowledge as central to community resilience, health sovereignty, and crisis adaptation. Future research should also examine the intersections between Indigenous motherwork and broader structural determinants of health—such as economic precarity, housing insecurity, and healthcare accessibility—to inform culturally responsive policies and interventions.

Moving forward, research should focus on expanding the scope of Indigenous maternal studies to better capture the complexities of motherwork in crisis contexts and beyond. Studies that take a community-driven approach and center Indigenous voices will be crucial in shaping policies and interventions that meaningfully support Indigenous mothers and families. Additionally, research should explore how Indigenous maternal knowledge can be integrated into public health and social support systems in ways that respect and uphold cultural sovereignty. By addressing these gaps, future scholarship can contribute to more inclusive and effective frameworks that recognize Indigenous motherwork as essential to both community well-being and broader societal resilience.

### Practice Implications

This study underscores Indigenous motherwork as both a critical caregiving practice and a form of resistance, particularly during the COVID-19 pandemic when systemic inequities placed disproportionate burdens on Indigenous mothers. The emergent themes—prioritizing survival needs, navigating oppressive forces, and embracing caregiving as both labor and leadership—demand a structural reorientation in how professionals, scholars, and policymakers engage with Indigenous maternal knowledge. Recognizing Indigenous motherwork as an epistemological, political, and survival strategy necessitates embedding it into healthcare, education, and policy frameworks to disrupt settler-colonial systems that undermine Indigenous maternal sovereignty.

For mental health practitioners and social service providers, the theme of prioritizing survival needs highlights the inadequacy of Western, individualistic models of care, which often fail to account for the gendered dimensions of Indigenous mothering. Indigenous mothers, as primary caregivers and household decision-makers were burdened with securing their families’ survival amid economic instability, healthcare inaccessibility, and increased caregiving responsibilities. However, Indigenous maternal labor is not only gendered but also deeply communal and intergenerational, resisting settler-colonial maternal paradigms that confine caregiving to the private sphere. Mental health interventions must move beyond Western nuclear-family models and integrate kinship-based and land-based healing practices that affirm Indigenous caregiving structures. Expanding Indigenous-led maternal mental health initiatives and incorporating culturally responsive therapeutic models that recognize caregiving as a relational and gendered political act is essential.

For educators and scholars, the theme of navigating oppressive forces exposes how colonial maternal ideologies have constructed Indigenous motherhood as either deviant or invisible. Settler-colonial policies have long targeted Indigenous mothers, policing their reproductive choices and fragmenting their families through surveillance, criminalization, and child removal. These processes are deeply gendered and racialized, reinforcing patriarchal, white supremacist logic that cast Indigenous women as either unfit mothers or self-sacrificing caregivers. The epistemic erasure of Indigenous motherwork in maternal studies, social work, and policy discourse upholds these colonial logics by privileging nuclear, patriarchal caregiving structures while dismissing Indigenous relational, interdependent, and reciprocal models of care. Scholarship must challenge these narratives by decentering Euro-Western maternal frameworks and engaging Indigenous intellectual traditions theorizing motherwork as a site of decolonial resistance and gendered political labor.

For activists and policymakers, the theme of embracing caregiving as both labor and leadership highlight the urgent need for structural policy interventions that recognize Indigenous maternal sovereignty and reject gendered colonial violence. The pandemic exacerbated preexisting inequalities, disproportionately burdening Indigenous mothers with increased unpaid labor while simultaneously restricting access to childcare, healthcare, and economic stability. Despite this, Indigenous caregiving practices remained inherently political, resisting neoliberal narratives of individualism and reinforcing caregiving as a form of community resistance. However, settler-colonial policies continued to obstruct Indigenous maternal autonomy through economic precarity, state surveillance, and punitive welfare policies that disproportionately removed Indigenous children from their families. Policy frameworks must move beyond crisis-driven responses and implement long-term, Indigenous-led maternal health, childcare, and economic justice initiatives that recognize Indigenous motherwork as foundational to both community well-being and self-determination. Dismantling patriarchal, colonial policies that surveil, criminalize, and fragment Indigenous families is not simply an act of equity but a necessity for Indigenous futurities.

Ultimately, Indigenous motherwork is a deeply gendered, political, and epistemological act that directly resists the patriarchal, colonial structures that have long sought to devalue and control Indigenous maternal labor. As this study demonstrates, Indigenous caregiving is not passive but an act of cultural survivance, political endurance, and intergenerational resistance. Failing to integrate Indigenous maternal knowledge into professional and policy frameworks not only reinforces colonial and gendered violence but also perpetuates the systemic erasure of Indigenous maternal sovereignty and resilience. Recognizing Indigenous motherwork as both a critical feminist and decolonial praxis is essential to dismantling settler-colonial legacies and ensuring Indigenous self-determination, gender justice, and the continued flourishing of Indigenous families and nations.

## Conclusion

This study affirms Indigenous motherwork as an essential practice of caregiving, cultural preservation, and resistance against settler-colonial systems that destabilize Indigenous kinship, sovereignty, and communal resilience. During the COVID-19 pandemic, Indigenous mothers navigated systemic neglect, balancing intensified caregiving burdens, economic precarity, and structural barriers while actively resisting colonial frameworks that impose nuclear family structures and pathologize Indigenous caregiving. Their labor, often dismissed or rendered invisible within dominant maternal paradigms, emerges as a critical site of epistemic authority, relational sustenance, and political resilience. Indigenous motherwork surpasses normative definitions of motherhood by asserting caregiving as a decolonial praxis rooted in intergenerational resilience, self-determination, and collective survival.

Recognizing Indigenous motherwork as a transformative, feminist, and anti-colonial praxis necessitates a structural reorientation within scholarship, policy, and governance. Interventions must move beyond crisis-driven responses to empower Indigenous leadership and embed Indigenous maternal knowledge within public health, social welfare, and governance frameworks. Rather than perceiving Indigenous mothers as passive recipients of intervention, they must be acknowledged as sovereign knowledge producers, architects of care, and agents of cultural and political renewal. Policy frameworks that uphold Indigenous maternal sovereignty and affirm intergenerational knowledge transmission are essential to disrupting colonial structures that continue to marginalize and police Indigenous caregiving. By prioritizing culturally responsive interventions and centering Indigenous knowledge systems, scholars and policymakers can contribute to the creation of futures rooted in justice, equity, and collective liberation, where Indigenous maternal knowledge is recognized not as ancillary but foundational to community resilience and well-being.
